# Supplementary material for: Overclaiming is not related to dark triad personality traits or stated and revealed risk preferences
Source: PLoS One. 2021 Aug 2;16(8):e0255207. doi: 10.1371/journal.pone.0255207 (PMC8328303; doi:10.1371/journal.pone.0255207)
Supplement: Attachment — Submitted filename: Response to Reviewers.pdf [file pone.0255207.s001.pdf]

Dr. Christiane Schwieren (Editor)

Comment E.1: *As you can see, both reviewers see that the study has value and contributes some innovative aspects to the literature. I agree with this general evaluation, and I also agree with the reviewers that you should be a bit clearer with respect to the relationship of your personality and behavioral variables. Both reviewers essentially indicate that there are some conceptual overlaps between overclaiming and overconfidence, but this might differ between individuals. The same holds for your dark triad factors. If you could be clearer in your arguments in this part, it would help to understand the contribution of the manuscript.*

Response: Thank you for the positive evaluation of the manuscript. In our revision of the manuscript, we tried to address all the questions you and the reviewers have raised. Please note that line numbers in our responses refer to the manuscript without tracked changes or the "simple markup" version of the revised manuscript (please see the responses to Comments 1.2, 1.3, 2.2, and 2.3.1).

Comment E.2: *A second point both reviewers discuss at differing lengths is your use of the measure of prenatal testosterone exposure. While reviewer one has quite some detailed questions about the measure, reviewer two makes a very important point - give a clearer argument why you need that measure at all.*

Response: We have incorporated both reviewers' suggestions into the manuscript. We now elaborate on why we have included this measure and why we think it is helpful to include it in the analyses (please see the responses to Comments 1.4.1, 1.4.2, 2.3.3, and 2.3.4).

Comment E.3: *There are also a couple of minor points in both reviews which you might want to consider.*

Overall, I think it is important especially in the case of a null-finding to give both a very good interpretation of it and a clear argumentation why an effect could have been expected. It must also be very clear that it is not an artifact of the methods applied or the sample size or other sample characteristics. If this is well done, a null finding is of course also interesting and publishable.

Response: We have considerably revised the manuscript to address all of the major and minor points made by the reviewers. We are confident we have arrived at an improved manuscript and are looking forward to your feedback.

Reviewer #1

Comment 1.1: *Summary & general evaluation*

On an undergraduate sample, the study examines the associations of overclaiming knowledge with the Dark Triad personality traits (i.e. subclinical forms of narcissism, Machiavellianism, and psychopathy), risk-preference, and other person-characteristics (e.g. gender, 2D4D ratio). The measurement methods applied are diverse including self-reported questionnaires and behavioural measurements. For example, a four-fold behavioural measurement method was used to assess participants' risk-preference. Overclaiming as the main dependent variable was operationalized with familiarity ratings given on 3 non-existing mathematical concepts. To analyse their data, the authors perform bivariate correlation and multiple regression analyses. The results did not support the hypothesized relationship of overclaiming with Dark Triad tendencies or stated and revealed risk-preferences.
The manuscript is clear, concise, and well-written. The systematic, and broad methodological approach is a high value of the paper. The data is analysed and reported properly. I have only a few comments / questions, please see them below.

Response: Thank you very much for your positive evaluation of our manuscript! We took great care to incorporate your thoughts and comments in revising the manuscript, and we hope you agree with us that the paper has improved. Please note that line numbers in our responses refer to the manuscript without tracked changes or the "simple markup" version of the revised manuscript.

Comment 1.2. *In the Introduction, the authors present the known associations of overconfidence with personality and behavioural traits and derive their hypotheses about overclaiming from the point that overconfidence and overclaiming are interrelated, although not completely overlapping concepts ("…overclaiming as one expression of overconfidence", line 336).*
From this point, however, it was surprising to me that analyses of associations of overconfidence with the personality and behavioural traits assessed were not performed by the authors. Of course, I understand that the paper is addressed to overclaiming, but considering that the two concepts (overclaiming and overconfidence) are so much related, and that overclaiming had no significant associations with the variables expected, it may help to interpret these negative findings if we see also the associations of overconfidence with the personality/behavioural traits measured in the study.

Response: We had not included (over-)confidence in the main analyses for the following reason: We did not assess actual mathematical aptitude in the present study. Therefore, we cannot say with certainty to which degree the stated confidence is actually overconfidence. To clarify this point, we have made several changes to the manuscript. For instance, we corrected a statement in the Procedure section saying that we had

adapted “an assessment of overclaiming and overconfidence.” We corrected the respective lines to say “confidence” instead of “overconfidence” as we used items similar to Shure et al.’s work. Still, we cannot disentangle confidence from overconfidence because we did not have an objective skill measure. Because of the observed correlation with overclaiming (as reported in the results section of our manuscript), we assume that at least some part of the confidence reported by the participants is, in fact, overconfidence. We have clarified this in lines 252 and 276 and added a new paragraph to the limitations section of the discussion to further explicate this issue. The new paragraph reads:

“*In contrast to the PISA assessment on which our confidence and overclaiming assessments are modeled [13], the present study lacks an objective assessment of mathematical aptitude. Therefore, we cannot tell whether participants’ confidence in the mathematical domain is justified or not without looking at overclaiming. Because overclaiming and confidence are meaningfully related (*r *= .301), we can at least say that some of the confidence reported by participants likely expresses overconfidence.*” (l. 431–435)

Comment 1.3: *The authors predict associations of overclaiming with Machiavellianism and psychopathy, mainly based previous findings showing a narcissism-overclaiming association. I agree that the associations of overclaiming with Machiavellianism and psychopathy are plausible, but the differences between the three Dark Triad traits may require a bit longer Introduction to understand the conceptual plausibility of these associations. For example, only a short sentence is provided for the possible association between psychopathy and overclaiming: “An association between psychopathy and overclaiming, in contrast, might indicate overclaiming instead to be more of an impulsive and less of a strategic act. (line 138)” One may expect a longer explanation of this potential association especially because Machiavellianism was also found to be related to self-reported and behavioural impulsivity (i.e. reward sensitivity); see e.g. Birkás, et al. (2015), Personality and Individual Differences, 74, 112-115.*

Response: Thank you for this comment and for pointing us to a highly relevant paper. We added the reference you suggested and two further references. We also extended our reasoning. The end of the paragraph now reads:

“*An association between psychopathy and overclaiming, in contrast, might indicate that overclaiming is more of an impulsive and less of a strategic act [33]. It is important to note that some previous research has identified links between concepts related to impulsive behavior and Machiavellianism [34] but other research has rendered conflicting results regarding such a link [33, 35].*” (l. 142–146)

We refrained from overextending this paragraph as Machiavellianism or psychopathy are not the focus of our research. As laid out in the original manuscript, our reasoning for including them was their associations with narcissism in popular questionnaires.

Moreover, we do not test a link between the personality traits of the Dark Triad and risk preferences, for which the work by Birkás et al., 2015, would probably make more robust predictions and would therefore be more relevant. Instead, we look at the Dark Triad traits only as antecedents of overclaiming. However, if you and the editor think that it is still warranted to include such inferences and suggestions, we will of course think about ways to add them to the manuscript.

Comment 1.4.1: *Methods. Digit ratio measurement was used to get an indirect anthropometric indication of the prenatal hormonal environment. The measurement and, also its limitations, are precisely described, only two questions emerged from my part. First, how many times the participants measured the length of their digits? Was it only a single-measurement, or multiple-measurement (e.g. three digit measures, and the mean is used for further analysis)? Second, was there any example image used to explain the correct digit measurement? For example, the length of digits is usually required to be measured from the basal crease of the digits to the tip of the digits. If this is not explained well, then it may be resulted in quite imprecise, and also inconsistent measurements across participants.*

Response: Thank you for highlighting this important point. We extended the description of the digit lengths measurement. It now reads:

"*We gave them a picture of a hand with an index finger stretching outward and a ruler displayed right below it. The distance between the tip of the index finger and its basal crease was highlighted and an explanatory sentence appeared below, giving the measurement of the index finger in the example picture. We subsequently asked them to use their ruler to measure the length of their right hand's second and fourth fingers once, as the right-hand digit ratio was found to be a better correlate of prenatal androgenization than the left-hand digit ratio [55]. In case participants had no ruler available, we also provided an on-screen ruler.*" (l. 259–265)

As you can see, in the text we now explicitly state that a single measurement was obtained and that participants were given an example picture highlighting the distance between the basal crease and the tip of the digit.

Comment 1.4.2: *Methods. Digit ratio was measured only on the right hand. I suggest the mentioning of the finding that right hand digit ratio is usually a more reliable indicator for testosterone exposure than the left hand ratio. See e.g.: Hönekopp, J. & Watson, S. Meta-analysis of digit ratio 2D:4D shows greater sex difference in the right hand. Am. J. Hum. Biol. 22, 619–630 (2010).*

Response: Thank you for bringing this up. We were building on this finding during survey creation and have now added the reference in the description of the measurement.

Comment 1.4.3: *Methods. Sample size calculation is provided, but could the authors give more details about their calculations? For example, how many predictors were used to calculate the a priori power of the regression analysis?*

Response: So far, we have provided a power analysis for finding bivariate correlations of a specific size (i.e., *r*s ≥ .212). We initially aimed for 200 participants, as this would have put us safely above 80% power to find correlations larger than .2. We found this to be reasonable compared to typical effect sizes in psychological research (Schäfer & Schwarz, 2019) and, for instance, the size of the gender effect on overclaiming in previous research (Jerrim et al., 2019). We clarified our approach in the materials and methods section of the manuscript. The relevant passage now reads as follows:

"*Before data collection, we aimed to recruit data from 200 participants as this sample size would be large enough to reliably (i.e., with 80% power) detect correlations of about* r = *.200, and we treated this as a threshold for effects large enough to be of interest. However, we missed this target due to dropout, but the actual sample size still allows for a reliable test (80% power) of correlations greater or equal to* r = *.214* [52]." (l. 241–244)

We did not conduct an a priori power analysis for the regression, as this was not the main analysis of interest for the present manuscript.

Comment 1.4.4: *Methods. As a quite valuable part of the study, four-fold measurement of risk preferences (one self-reported measure, and 3 gamified behavioural measures) was assessed. In contrast, however, the most important dependent variable of the study – overclaiming – was tested only by three questionnaire items which specifically related to mathematical problems. I agree that this may be an "elegant and fast way to assess" (line 433) overclaiming, but I feel it a bit a minimalist approach (especially in contrast with the systematic of risk-taking assessment). Could the authors elaborate that how widely this operationalization method was used in previous studies?*

Response: We added a sentence and three references using this approach (and the same number of foils) to the description of the measurement in the discussion section. It now reads:

"*Second, overclaiming in the present study meant to rate nonexistent math concepts as familiar. More specifically, overclaiming was measured by participants' answers to three questionnaire items. While this represents a commonly used, elegant, and fast way to assess a concept as fuzzy as overclaiming [13, 63, 64], it also amounts to cheap talk that bears minimal risk of any negative consequences for participants.*" (l. 455–458)

Comment 1.4.5: *Methods. Cronbach's α for the Dark Triad subscales are rather low. It may be meaningful to show the McDonald's omega in addition to the alpha.*

Response: We followed your suggestion and added McDonald's ω for all scales reported in Table 1.

Comment 1.5: *Results and Discussion are quite well written; the authors elaborate many implications of their findings. I have no questions regarding these sections, in this first manuscript version.*

Response: Thank you again for your thoughtful and considerate comments and questions that helped us revise the manuscript. We hope to have addressed them to your satisfaction.

Reviewer #2

Comment 2.1: *This is a detailed manuscript reporting the results from an investigation of the relationship between overclaiming one's knowledge (conceptualized as a facet of overconfidence), gender, personality traits and risk preferences. In order to measure overclaiming experimentally participants were instructed to solve a set of various math problems and their familiarity with a set of math concepts some of them being not existent. Risk preferences were measured by further three behavioral tasks, and digit ratio was included as a measure of testosterone influence during prenatal development. The whole study was performed online. Contrary to their hypothesis overclaiming was found not to be related to risk preferences or the personality traits investigated.*

Response: Thank you for your willingness to review our manuscript and for the many thoughtful comments you have provided. We took great care to respond to your comments adequately. We hope that our revised manuscript will be to your satisfaction. Please note that line numbers in our responses refer to the manuscript without tracked changes or the "simple markup" version of the revised manuscript.

Comments 2.2: *A rather broad introduction focuses on the relevant literature on overconfidence thereby following the assumption of a major conceptual overlap between overconfidence and overclaiming. Notably, although there is a significant correlation between overclaiming and self-confidence in the present study, a correlation of r = .301 does not reflect a high conceptual overlap between both concepts questioning the starting point of this ms. that is "A phenomenon that …can be classified as a behavioral expression of overestimation is the tendency to overclaim" (page 3 line 42/43). The assumption of a high conceptual overlap also constituted the selection of tasks dealing with financial risk taking and personality traits. However, overclaiming may reflect overconfidence in some individuals but may have quite other sources in many individuals as stated by the authors themselves on the top of page of 19.*

Response: Thank you for bringing up this important point, which is related to Comment 1.2 of Reviewer 1. Unfortunately, we did not assess actual mathematical aptitude in the present study, and, therefore, we cannot say with certainty to which degree the stated confidence reflects overconfidence. Accordingly, the correlation of .301 pertains to the link between confidence and overclaiming rather than to the link between overconfidence and overclaiming and does not necessarily reflect a lack of overlap between the latter concepts.

We corrected a line in the procedure section to say "confidence" instead of "overconfidence" as we use items similar to Shure et al.'s work but cannot fully disentangle confidence from overconfidence in this measure because we did not have an objective skill measure. Only because of the correlation between our measures of confidence and overclaiming are we confident to say that at least some part of the reported confidence is, in fact, overconfidence. We have clarified this in lines 252 and

276 and added a small paragraph to the limitations section of the discussion to further address this issue. The new paragraph reads:

"*In contrast to the PISA assessment on which our confidence and overclaiming assessments are modeled [13], the present study lacks an objective assessment of mathematical aptitude. Therefore, we cannot tell whether participants' confidence in the mathematical domain is justified or not without looking at overclaiming. Because overclaiming and confidence are meaningfully related (r = .301), we can at least say that some of the confidence reported by participants likely expresses overconfidence.*" (l. 431–435)

To rephrase, we have assessed confidence in the mathematical domain, of which some part is probably in line with the participants' actual mathematical aptitude. However, some of it might reflect overconfidence, as indicated by the correlation with the overclaiming measure. This assumption (and our assessments of confidence and overclaiming) is based on previous research (Jerrim et al., 2019) where the authors found a substantial correlation between overclaiming and overconfidence by adjusting their confidence measure for gender, socio-economic status, immigrant status, PISA scores for reading, math, and science, and school effects. This shows that some of this confidence is overconfidence not justified by the individuals' aptitude. Participants who overclaim were significantly more confident on every single item of the confidence measure (see Table 3 of Jerrim et al., 2019).

Comments 2.3: *Some further questions are arising from the selection of tasks and personality measurements:*
Comment 2.3.1: *E.g. although the authors on page 5 cite several studies that suggest no meaningful correlations between overclaiming and narcissism the selection of the Dark Triad as psychometric measurement of personality traits suggest that the authors expected narcissism to be related to overclaiming not considering that narcissism is often based on motives such as hiding one's fragile self-worth rather than overconfidence.*

Response: We are sorry for having caused confusion with our line of arguments. It is true that we introduce a potential link between narcissism and overclaiming by bringing up conflicting results with two studies reporting a significant relationship and two studies (with larger numbers of participants but less precise assessments of narcissism) that suggest no meaningful relationship. Your comment provides a pertinent link between these two sets of results, so we added a corresponding sentence and two references to address the issue you raise. It now reads:

"*Regarding personality traits, overclaiming has been initially linked to self-esteem and narcissism as a form of self-enhancement and socially desirable response pattern [10]. Both links make sense intuitively, not only because narcissism and overconfidence are also reportedly linked [27] but because they provide an answer to the motivational*

*question of why people tend to overclaim in the first place. Overclaiming makes people feel better about themselves and presents an opportunity to show others how sophisticated, well-read, competent, or adventurous one is. However, some facets of narcissism are also based on motives like protecting one's fragile self-worth [28, 29]. Consistent with this complication, two research efforts are muddying the evidence regarding the relationship between narcissism and overclaiming. One is a large-scale online study with over 1,500 participants in which the authors observed a positive correlation between narcissism and overclaiming that was statistically significant but of negligible size (*r *= .12) [30]. The other is a series of four studies with a total number of 1,300 participants, also reporting no meaningful correlation between overclaiming and a personality factor related to narcissism [31]. Thus, these results strongly suggest that the link between narcissism and overclaiming may be weaker than initially assumed.*" (l. 118–130)

Comment 2.3.2: *Page 8: As the authors write, the trust game according to a recent meta-analysis may investigate social preferences like prosocial behavior rather than providing information about social risk taking.*

Response: Thank you for bringing this up. However, note that prosocial behavior and social risk taking might not be mutually exclusive at all. To trust someone by cooperating in the Trust Game is both a prosocial behavior and a risky one. This trust can be broken. It is true that not investing (i.e., not trusting the trustee) could be motivated by a) competitive individualism, b) risk aversion, or c) a combination of the two. Therefore, it is true that a prosocial orientation might motivate the initial tendency to cooperate in the Trust Game; however, it is still the individual's risk propensity whether to go through with it.

We added a sentence that clarifies our understanding of the interplay between social and risk preferences regarding the Trust Game in the Introduction. The relevant passage now reads: "*A recent meta-analysis of commonly studied economic games identified over 400 individual studies using investor decisions in the Trust Game and established that it is primarily used to investigate social preferences like prosocial behavior [51]. Keeping this in mind, social preferences might be an initial driver of the decision to invest or not, but individuals' risk preferences then play a role in whether to go through with investing. Therefore, the Trust Game perfectly complements the BART and the Investment Task's rather general and monetary-focused assessments, respectively.*" (l. 203–208)

Comment 2.3.3 – *Page 16, 369: Why do the authors control for the influence of digit ratio when they are searching for gender effects?*

Response: As previous research suggests, associations between testosterone concentration and digit ratio can be observed independent of gender. As elevated

testosterone levels have been assumed to be a potential cause of overconfidence, we were interested in adding the digit ratio as a biologically determined measure of prenatal androgenization, along with the socially and culturally determined dichotomy of gender. That said, note that our analysis permits a separate evaluation of the effects of digit ratio and gender.

We added a sentence to the Introduction highlighting this reasoning. This sentence reads: "*We included both digit ratio and gender in the present study to be able to disentangle influences that are perhaps primarily driven by cultural and social norms and expectations (i.e., gender) from more biological determinants of behavior (i.e., assessed via the digit ratio).*" (l. 115–117)

Comment 2.3.4: *Page 15: please insert "digit ratio as marker of prenatal testosterone"*

Response: We inserted the specification with a slight modification that aligns it with how the digit ratio is described earlier in the manuscript: "*digit ratio as a correlate of prenatal androgenization*" (l. 378f)

Comment 2.3.5: *I am wondering whether math knowledge and abilities have influenced the results of a very specific and restricted assessment of overclaiming. However, education and knowledge have not been controlled for as far as I see.*

Response: Assessments of education (e.g., school years) and knowledge (i.e., an objective assessment of mathematical aptitude) were not part of the survey as the participants were primarily students who, empirically speaking, do not differ much by education. We did not include proxies for mathematical aptitude (e.g., the last school grade in mathematics), but we included the students' major, which allowed us to test whether the results differ if we, for example, exclude students with a math-heavy economics major. Doing so did not lead to different results. Please also see our response to Comment 2.2 regarding the results of Jerrim et al. (2019).

Comment 2.4: *In sum, this is a somehow innovative study following an important question which gains special interest in times of a growing amount of fake news. However, I was not surprised that the authors could not confirm their hypotheses since the rationale for their studies is not guided by a stringent understanding of the concepts they intended to investigate.*

Response: Thank you for your evaluation and your helpful comments. Concerning the rationale of our study, we want to reiterate that we built our hypotheses on the established link between overconfidence and overclaiming (Jerrim et al., 2019). As we stated in response to your Comment 2.2, the relatively moderate correlation between confidence (that we had misleadingly depicted as overconfidence at some places of the manuscript) and overclaiming does not conflict with this assumption. We hope that clarifying this vital point–along with our other revisions, responses, and arguments–makes our rationale more traceable and highlights that our findings advance research on overclaiming.

References

Schäfer T, Schwarz MA. The meaningfulness of effect sizes in psychological research: Differences between sub-disciplines and the impact of potential biases. Front. Psychol. 2019;10:813 doi: 10.3389/fpsyg.2019.00813

Jerrim J, Parker P, Shure N. Bullshitters. Who are they and what do we know about their lives? IZA Discussion Papers. 2019. Available from: https://www.iza.org/publications/dp/12282/bullshitters-who-are-they-and-what-do-we-know-about-their-lives
